# Longitudinal deterioration of white-matter integrity: heterogeneity in the ageing population

**DOI:** 10.1093/braincomms/fcaa238

**Published:** 2021-01-22

**Authors:** Konstantinos Poulakis, Robert I Reid, Scott A Przybelski, David S Knopman, Jonathan Graff-Radford, Val J Lowe, Michelle M Mielke, Mary M Machulda, Clifford R Jack, Ronald C Petersen, Eric Westman, Prashanthi Vemuri

**Affiliations:** 1 Department of Neurobiology, Care Sciences and Society, Karolinska Institutet, Stockholm 141 52, Sweden; 2 Department of Radiology, Mayo Clinic, Rochester, MN 559 05, USA

**Keywords:** white-matter microstructure, cognitive decline, longitudinal clustering, resilience

## Abstract

Deterioration in white-matter health plays a role in cognitive ageing. Our goal was to discern heterogeneity of white-matter tract vulnerability in ageing using longitudinal imaging data (two to five imaging and cognitive assessments per participant) from a population-based sample of 553 elderly participants (age ≥60 years). We found that different clusters (healthy white matter, fast white-matter decliners and intermediate white-matter group) were heterogeneous in the spatial distribution of white-matter integrity, systemic health and cognitive trajectories. White-matter health of specific tracts (genu of corpus callosum, posterior corona radiata and anterior internal capsule) informed about cluster assignments. Not surprisingly, brain amyloidosis was not significantly different between clusters. Clusters had differential white-matter tract vulnerability to ageing (commissural fibres > association/brainstem fibres). Identification of vulnerable white-matter tracts is a valuable approach to assessing risk for cognitive decline.

## Teaser

White-matter health deterioration plays a role in cognitive ageing. Using longitudinal imaging data, Poulakis *et al.* found four heterogeneous clusters of individuals with distinct spatial patterns of white-matter integrity trajectories. This heterogeneity was related to systemic health and not to brain amyloidosis and was associated with distinct cognitive trajectories.

## Graphical Abstract

**Graphical Abstract:**
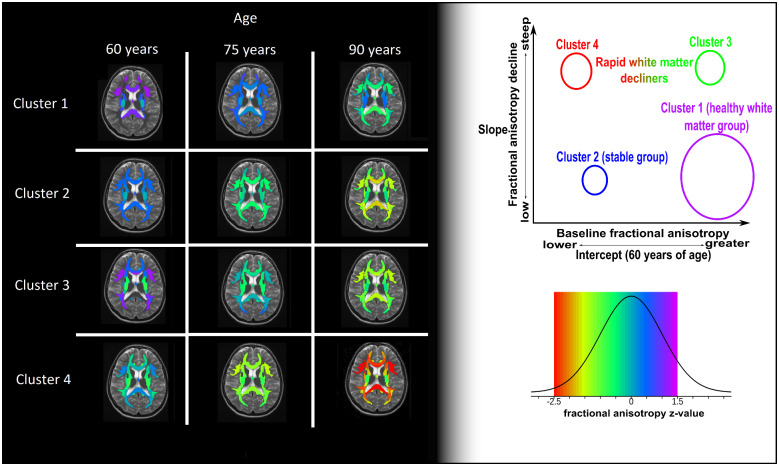

## Introduction

White matter (WM) changes are widely found in the elderly as part of the ageing process, as well as pathological processes. Although white-matter hyper-intensities (WMH) are often investigated as predictors of poor cognitive and motor function, there is evidence for early changes in WM even before the appearance of WMH (Maillard *et al.*, 2012; Wardlaw *et al.*, 2015). These early changes can be captured by changes in microstructural integrity assessed using diffusion tensor imaging (DTI) (Croall *et al.*, 2017) measures such as fractional anisotropy (FA) which allows the estimation of coherence of WM tracts through the quantification of the water-diffusion properties. The vast literature on DTI has consistently shown associations between cognitive and motor function with ageing (Kennedy and Raz, 2009; Madden *et al.*, 2009). While DTI articles have been focussed on specific tracts to investigate disease-specific hypotheses, e.g.—hippocampal DTI signal in vascular dementia in (Ostojic *et al.*, 2015), there have been other articles that have broadly investigated regional WM micro-structural differences with age (Burzynska *et al.*, 2010).

Cohort studies incorporating serial high-quality diffusion MR along with detailed subject-specific disease information (brain amyloidosis, health-care records and cerebrovascular disease) have recently become mature. This longitudinal imaging data across the age span provides us with an opportunity to investigate early WM changes and assesses how different tracts may progress differentially with ageing in a data-driven manner without specifically looking at a smaller number of tracts. In the literature, single tract-based approaches have been used typically in which each tract is analysed separately (Barrick *et al.*, 2010; Charlton *et al.*, 2010; Sullivan *et al.*, 2010; Teipel *et al.*, 2010; Moscufo *et al.*, 2018; Molloy *et al*., 2019; Rabin *et al.*, 2019). These studies provided us with important age-related tract-level inferences. However, understanding of differential ageing of WM tracts over time has significant biological implications. This is not feasible with univariate tract-based solutions because they do not consider the correlation between tracts over time and are unable to completely detect the multivariate associations. The recent advances in multivariate unsupervised statistics have made the longitudinal analysis of WM integrity throughout the brain possible. Using these new methods, we are now able to incorporate longitudinal information from many WM tracts simultaneously in order to discover and visualize clusters of individuals, while accounting for confounders whose effects need to be accounted for.

In this study, we hypothesized that a data-driven investigation of changes in longitudinal WM integrity throughout the brain (using multivariate approaches) may provide unique insights into the ageing process and improve our understanding of the impact of WM integrity on cognitive outcomes. Here, we leveraged longitudinal DTI data from the population-based Mayo Clinic Study of Aging (MCSA), to construct the trajectories of WM integrity among participants aged 60 years and older. The specific goals of this article were to discover clusters of study participants with different WM integrity trajectories, and to investigate the factors that associate with the spatial distribution of decline in their WM. We hypothesize that the integrity of specific WM tracts may help separate individuals with diverse profiles of future cognitive decline.

## Materials and methods

### Selection of participants

The participants of this study were enrolled in the MCSA. This is a population-based study of Olmsted County, Minnesota, residents. The Rochester Epidemiology Project medical records linkage systems were used for the enumeration of the Olmsted County population. Details of the MCSA have been described previously (Petersen *et al.*, 2010). Standard protocol approvals, registrations and patient consents: The Institutional Review Boards of the Olmsted Medical Center and Mayo Clinic approved the study, and written informed consent was obtained from all participants. We included all 553 elderly individuals (age, ≥60 years) with at least two DTI imaging scans (Supplementary Fig. 1). At the time of the baseline scans, 486 were cognitively unimpaired (CU), 62 had mild cognitive impairment, 4 were diagnosed with a neurodegenerative disorder (3 Alzheimer’s clinical syndrome with dementia and 1 with vascular dementia) and 1 had a missing clinical diagnosis due to incomplete data. A strength of the study is the population-based nature of the cohort. By using a broader range of individuals with cognitive problems in the population, we are positioned to detect the most important WM changes seen in the ageing population. All participants had at least two imaging visits, 125 had at least three visits, 11 at least four visits and finally 1 participant with five visits. The time between visits/intervals (Supplementary Table 4) was accounted for in our analyses. Moreover, the characteristics of the patients who dropped out from the study (only one visit) were not statistically different from those who were followed up. However, some cognitive domains and functional abilities were slightly altered (Supplementary Table 5). Age at the time of MRI scan, sex and *APOE4* genotype were considered for this study. We also included a summary measure for cardiovascular and metabolic conditions (CMC) ascertained from electronic health records in the 5 years prior to the MRI (Vemuri *et al.*, 2017). The main characteristics are summarized in Table 1.

**Table 1:** Demographics of the cohort

Age at the baseline scan	60-70	70-80	80+
Age group characteristics			
*N*, *N* (%)	247 (45.00%)	188 (34.00%)	118 (21.00%)
Males, *N* (%)	128 (51.80%)	98 (52.10%)	74 (62.70%)
Age, median (mad)	65.00 (3.00)	74.90 (3.70)	84.50 (4.10)
Education (years), median (mad)	16.00 (2.97)	14.00 (2.97)	14.50 (3.71)
APOE4 allele carrier, *N* (%)	71 (28.70%)	59 (31.40%)	32 (27.10%)
APOE2 allele carrier, *N* (%)	40 (16.2%)	32 (17%)	12 (10.20%)
Health, mean (SD)	2.20 (0.90)	2.10 (0.80)	2.30 (0.90)
Baseline_CMC, mean (SD)	1.70 (1.20)	2.00 (1.30)	2.90 (1.40)
Gait speed, median (mad)	121.00 (15.86)	114.00 (18.09)	100.70 (20.76)
WMH/TIV × 100, median (mad)	0.38% (0.24)	0.82% (0.54)	1.44% (0.91)
SPM12_PIB_RATIO, median (mad)	1.39 (0.09)	1.41 (0.15)	1.56 (0.32)
Diagnosis and cognition			
CU, *N* (%)	230 (93.10%)	161 (85.60%)	95 (80.50%)
Global, median (mad)	0.47 (0.83)	0.05 (0.98)	−0.34 (0.94)
Memory, median (mad)	0.45 (0.91)	0.00 (1.15)	−0.36 (1.40)
Executive, median (mad)	0.38 (0.77)	−0.02 (0.80)	−0.50 (0.99)
Language, median (mad)	0.41 (0.90)	−0.04 (1.00)	−0.25 (0.88)
Visuospatial, median (mad)	0.38 (0.85)	0.12 (0.86)	0.05 (0.93)

Main demographic, clinical and biomarker characteristics of the data set. Health, how healthy the participant feels from 0 to 4 with 4 being worst; CU, cognitively unimpaired; WMH/TIV × 100, WMH in T2 MRI as a fraction of total intra-cranial volume; SPM12_PIB_RATIO, Pittsburgh compound B (PiB) PET SUVR; mad, median absolute deviation. The last five domains that start with PZ refer to cognitive functionality; they are normative *Z*-values and higher values correspond to better cognitive score.

### Diffusion tensor imaging

All DTI images were acquired on three 3T GE MRI scanners (GE Medical Systems, Milwaukee, WI) at Mayo Clinic, Rochester. The DTI acquisition protocol was a 2.7-mm isotropic resolution spin echo sequence with five *b* = 0 volumes followed by 41*b* = 1000 s/mm^2^ diffusion weighted volumes with directions evenly spread over the whole sphere. The data was processed as discussed earlier (Vemuri *et al.*, 2018). After the calculation of the diffusion tensors, FA and mean diffusivity images were computed. Voxels with mean diffusivity of >2 × 10^−^^3^ or <7 × 10^−^^5^ mm^2^/s were excluded as mostly CSF or non-brain tissue, respectively. Then an atlas was registered by warping its template FA image to each participant’s FA using ANTS software (Avants *et al.*, 2008). The atlas was a modified version of the Johns Hopkins ‘Eve’ atlas. The only modification to the atlas was to combine the left and right halves of structures spanning the medial plane, such as the genu and pons (Oishi *et al*., 2009). Regions of interest with <7 voxels in subject space were excluded as too small to be reliably registered. Finally, the median FA and mean diffusivity in each region were computed. Regions with very small FA values (<0.25) consisted of mostly grey matter or grey-matter/WM boundaries (caudate nucleus, amygdala, globus pallidum, hippocampus, putamen, thalamus, entorhinal area and lingual WM) and therefore were excluded. Participants with many missing values were excluded as registration errors. Because of the greater sensitivity of FA to subtle microstructural changes in comparison to mean diffusivity, we focussed on FA in this study (Burzynska *et al.*, 2010; Molloy *et al*., 2019). Forty-eight FA measurements were used in the analysis. Of them, 20 were considered superficial WM and 28 were deep WM tracts (Supplementary Table 2). No differences between MRI scanners were found in the FA metrics (Supplementary Table 3).

### Ascertainment of pathologies from imaging

#### Amyloid load from Pittsburg compound B–PET

The acquisition, processing and summary measure details for amyloid PET on the MCSA study participants have been discussed previously (Jack *et al.*, 2017). For amyloid PET, the global amyloid load was computed for each participant by calculating median uptake in the pre-frontal, orbitofrontal, parietal, temporal, anterior cingulate and posterior cingulate/precuneus regions of interest divided by the median uptake in the cerebellar crus grey-matter regions of interest. The cut point for normal/abnormal amyloid PET was considered as defined from Jack *et al.* (2017) and was a standardized uptake value ratio of 1.48 (centiloid 19).

#### White-matter hyper-intensities from FLAIR–MRI

Brain infarctions were assessed by trained image analysts and confirmed by a clinician blinded to all clinical information. WMH were ascertained using an in-house semi-automated method which is based on clustering via connected components that are then masked to remove high likelihood grey-matter voxels as described in detail by Graff-Radford *et al.* (2019). These detected WMH masks were edited for errors by trained image analysts and infarctions were removed from the WMH estimation.

### Cognitive performance

The MCSA neuropsychological battery, as previously described, covers four cognitive domains with nine tests (Roberts *et al.*, 2008). In this analysis, we used the four domain-specific *Z*-scores: executive function (Trail-Making Test: Part B, Wechsler Adult Intelligence Scale-R Digit Symbol); visuospatial performance (WAIS-R Picture Completion, WAIS-R Block Design); memory [Wechsler Memory Scale (WMS)-R Visual Reproduction-II (delayed recall), WMS-R Logical Memory-II (delayed recall) and Auditory Verbal Learning Test delayed recall]; language (Boston Naming Test, category fluency) and a global *Z*-score which was the average of all domain scores transformed to *Z*-scores.

### Statistical clustering

In this study, we aimed to find groups of individuals with similar longitudinal FA profiles. We applied the longitudinal cluster analysis framework that we developed recently for neuroimaging measures (Poulakis *et al.*, 2020) on the FA measurements of 48 WM regions from the JHU atlas described in the DTI methods section. Briefly, our approach is based on generalized mixed-effect model and Gaussian mixture modelling for clustering. We estimate each participant’s longitudinal FA trajectory (intercept and slope) using the mixed-effect model. Then, we compare the trajectories across all participants and clustered individuals according to the similarity in intercept and slope using Gaussian mixture model. Our framework simultaneously accounts for irregular sampling and an unequal number of images per individual; accounts for confounding effects; provides cluster visualization and measures clustering uncertainty as described in detail by Poulakis *et al.* (2020). The R language implementation that was used has been explained by Komárek and Komárková (2014). We controlled for sex and *APOE4* carrier status (six patients were excluded because of missing data). The random effect (variable to account for repeated measurements in time in the study) was years from the age of 60 years (e.g. the variable takes the value of 5 for a participant who was 65 years old at the age of the baseline MRI). This time variable is a continuous measure that can help in the interpretation of the clusters.

#### Model implementation

The initialization of the algorithm was repeated five times and for seven different cross-sectional cluster solutions (two to eight cluster models) to find the most optimal model and explore the data. More specifically, we applied random forest clustering and four variants of a Gaussian mixture model (Bergé *et al.*, 2012) on the baseline data to find good values for the initialization of the longitudinal clustering. We selected the best model based on three criteria of model quality: deviance [−2 × log(model likelihood)] of the model, Markov Chain Monte Carlo iterations convergence (parameter samples first lag auto-correlation) and number of participants with uncertain cluster allocation (based on highest posterior density intervals). In total, we trained 35 models (five initializations times seven different cluster numbers), for 250 000 Markov Chain Monte Carlo including the burn in. After the model evaluation, we inspected the classification probability matrix. We provide results for the best model in this article.

### Relationship between WM tracts and cluster characteristics

After cluster identification, our goal was to examine the regions that discriminated between clusters of the model output. We used two different methods to aid in the process. First, we estimated the cluster/region mean intercept (age, 60 years) and slope (presented as annual change) for the different types of fibre tracts. This provided us with a straightforward visual method to extract discriminative features between clusters of WM integrity. Second, we also employed generalized linear mixed-effect models (number of clusters − 1 logit models) with shrinkage priors (Piironen and Vehtari, 2017; van Erp *et al.*, 2019) to investigate which regions were most discriminative between all the identified cluster combinations. Those regions were defined with a ranking system of odd-ratio probabilities. For each of the models, we selected regions that separated pairs of clusters (at the baseline and/or over time) with at least 60% of probability. In this way, we sorted out the most irrelevant regions. Then we combined the results of all models (number of clusters − 1 logit; e.g. three clusters produce two models) and selected the final set of discriminant regions based on the regions that were common between models or very important for the separation between a pair of clusters. Based on this technique, we identified discriminant regions that are important for capturing both heterogeneity between WM integrity clusters and also those WM tracts that deteriorate with ageing in the study population. Regional FA was used as input and cluster allocation as output and therefore a logit link represented the categorical cluster allocation. These two different methods allowed us to confirm the results of the visual approach (clustering output) agreed with those from the supervised approach (post-clustering comparison).

### Features that differentiate participants of FA clusters

After extracting the regions that were highly discriminative between clusters, we added a final supervised model in our analysis to assess the regional WM integrity—cluster relationship with other factors. We trained a multi-variate multi-output mixed-effect linear regression. Cluster allocation as well as demographic characteristics and biomarker values were used as input and the regional FA discriminant variables as output. This model estimated the extent to which clusters may differ in characteristics other than WM integrity distribution trajectories.

### Data availability

Data that supports the findings of this study is available upon reasonable request from the MCSA investigators.

## Results

The characteristics at the time of the baseline MRI scan are summarized in Table 1 as a function of age decades. As expected, we found that increasing age was associated with worse cognition and gait speed, greater amyloid burden, WMH and a greater number of CMC.

### Statistical clustering and classification of subjects

**Figure 1:**
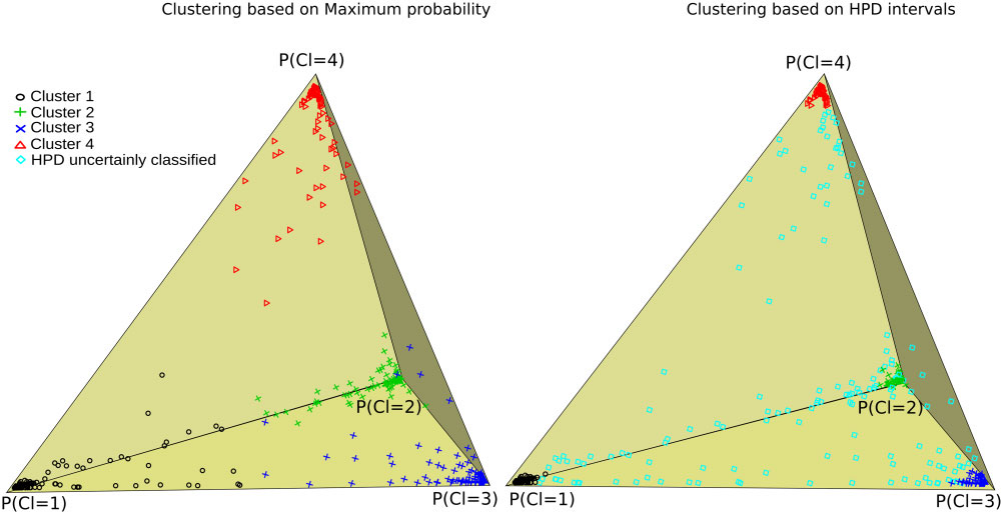
**Cluster–subject probability classification.** The subject–cluster allocation matrix is visualized with the help of multidimensional scaling. The closer a subject (dot) is to one of the pyramid corners the higher is the chance that the participant belongs to the cluster expressed by that figure side. The classification of subjects is based on the maximum probability rule (left figure) and HPD intervals (right figure) to assess uncertainty in the classification. The two figures differ only in the colouring of the uncertainly classified subjects (cyan blue colour). We observe that (i) most of the participants are on the base of the pyramid, (ii) between clusters 1 and 4 there are few cases, whereas (iii) between clusters 1–2 and 1–3 more cases exist. This translates to the similarities between clusters in terms of WM integrity.

We evaluated models by the number of total clusters. The model assessment showed that for each number of clusters, one model was clearly better than the rest with similar parameters but different initial parameter values. While the optimized model with two clusters received the highest quality scores (lowest model deviance, fewer subjects with uncertain classification and fewer Markov Chain Monte Carlo chains with poor mixing), it separated only the participants with high FA values from participants with low FA values, which was not informative. We did not choose this model, because it separates the sample by means of progression which does not add any new information and was not considerably different from the next solution (four clusters) in terms of quality (Supplementary Table 1). We include the solution for two clusters in Supplementary Tables 6 and 7 and Supplementary Fig. 3. The next best solution was the one with four clusters, which was chosen for interpretation. This model had four FA components and the participants who were clustered together in each cluster were decided based on the estimated highest posterior density intervals (Fig. 1). A highest posterior density interval is interpreted as the Bayesian modelling equivalent to confidence interval in cases where samples from posterior distributions exist to estimate parameter error. The clustering based on the maximum probability of each participant belonging to any cluster is shown in Fig. 1 (left).

A low representation (first three principal components of the multi0dimensional scaling plot for the component–subject probabilities) of the cluster allocations shows that cluster 1 and cluster 4 are very different, whereas clusters 2 and 3 are more similar to each other (Fig. 1, left). The cyan colour data points shown in Fig. 1 (right) represent participants who were not clustered to any of the cluster/components with high certainty. A total of 143 participants were excluded from the cluster demographics table since they were not grouped to only one cluster with high certainty (intermediate) and add noise to the interpretation of the four distinct groups of clusters. However, the contribution of the intermediate participants was crucial to understand which clusters are similar in terms of WM integrity.

### Interpretation of the clusters

**Figure 2:**
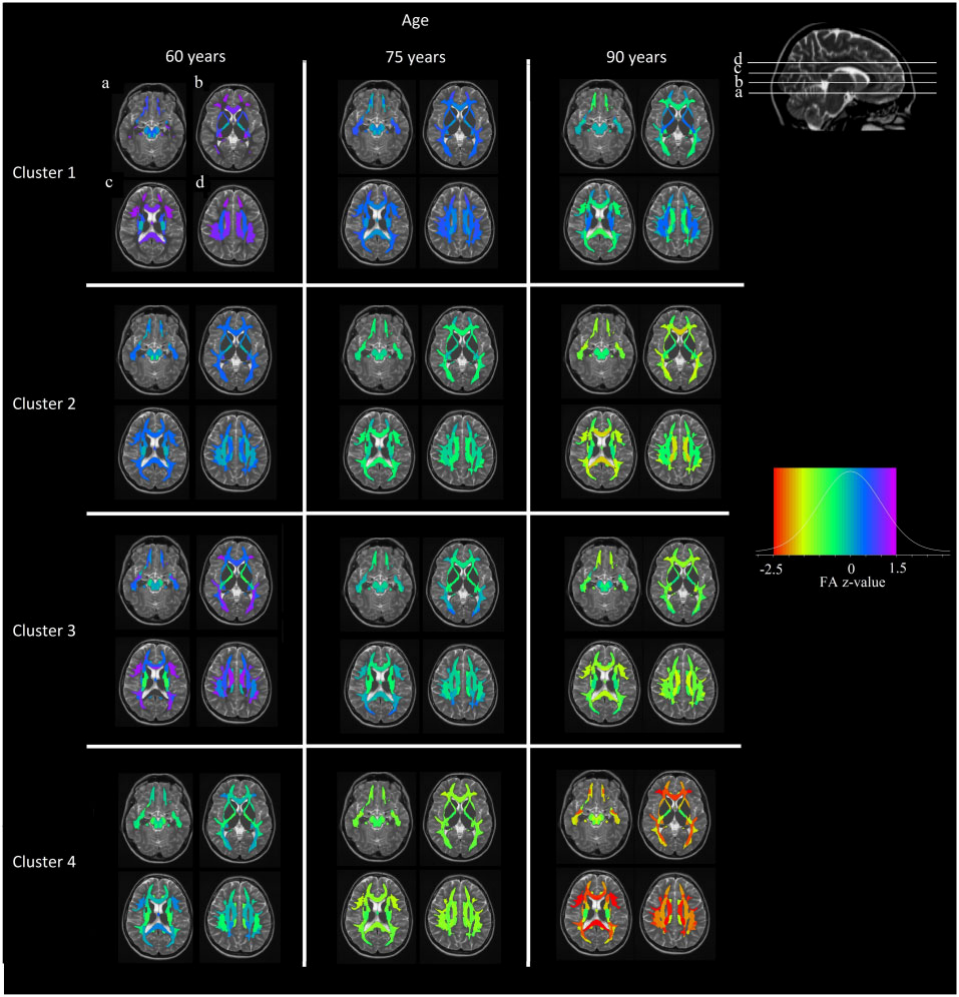
**Cluster-specific WM integrity profiles at different ages.** Clustering model-fitted values controlled by sex and APOE4 carriership. Fitted values were calculated for the age of 60 years (model intercept), 75 and 90 years. The red colour at the left of the colour legend shows lower FA in comparison to yellow colour. The data is *Z*-value transformed. The colour scale legend includes five colours ordered by increasing FA (red, yellow, green, blue and purple). Clusters are sorted in terms of WM integrity severity trajectories, whereas other trajectories exist too.

As shown in Fig. 2, we provide the model-fitted values for the clusters of four different FA profiles over time and for fixed effects (sex = male and APOE4 = negative). We have ordered the cluster numbers based on WM health at baseline and the rate of WM declines. Cluster 1 had the highest baseline FA overall in all types of fibres followed by clusters 2 and 3. Cluster 4 had the lowest FA levels among the clusters at the baseline estimation (Fig. 2; age, 60 years). Cluster 1 showed a stable decline in WM integrity over time, which was much slower than for the rest of the clusters. Cluster 4 shows a stable deterioration for some fibres and very steep decline for some others, reaching the lower end of the FA values observed in the data set (Fig. 2, red colour in legend). Clusters 2 and 3 have some similarities in the baseline-estimated FA values, whereas they have very different progressions over time. Cluster 2 has lower FA values than cluster 3 for most fibres, since at baseline cluster 3 starts with high FA values for some association fibres while cluster 2 does not. However, with time cluster 3 quickly approaches the WM integrity of cluster 2 for all fibres, if not lower FA in some regions.

**Figure 3:**
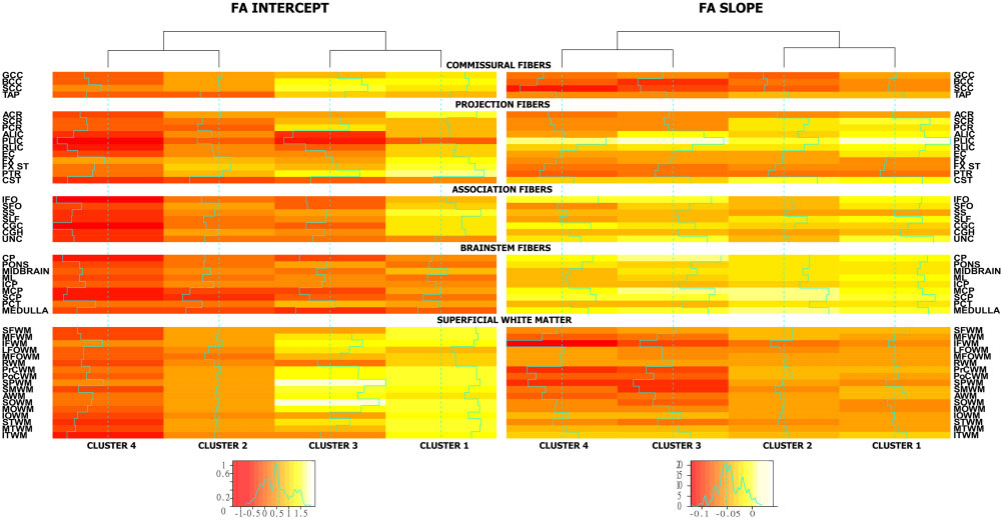
**Cluster intercept and slope mean FA coefficients.** The two different heat maps present the estimated mean intercept and slope for the FA *Z*-values over the variables that were used in the clustering. The intercept spans values between −1 and 1.5, whereas the slopes are mostly negative due to the decline in FA over time. Different colour scales are used for the slope and intercept. The values used are estimated output of the clustering model. Clusters are grouped into two pairs where clusters with most similar profiles are together (see dendrogram above heatmaps). Clusters 2 and 3 are similar in intercepts, whereas clusters 2 and 4 are similar in slopes (FA decline over time).

Regarding the proportions of participants in the clusters, cluster 1 has the highest number (*n* = 186, 45.4%) in the sample, followed by cluster 4 (*n* = 87, 21%), cluster 3 (*n* = 74, 18%) and finally cluster 2 (*n* = 63, 15.5%). No substantial differences were observed in sex between the clusters, whereas females had systematically lower FA values in a number of regions. Differences were not observed in *APOE2* or *APOE4* carriership status across the clusters. Approximately, 90% of individuals in clusters 1 and 3 were CU at baseline, cluster 2 follows with 84% and cluster 4 has the lowest percentage (82%) of CU individuals (Table 2). Gait speed was not considerably different among clusters, but cluster 4 had lower gait speed and higher median absolute deviation. Cluster 4 has the lowest estimated FA values at the intercept (age, 60 years), followed by cluster 2 with similar or better values among the sum of fibre tracts. Cluster 3 follows with higher estimated values in commissural fibres, similar in association fibres to cluster 2 and finally different in projection fibres compared to both clusters 2 and 4. Finally, cluster 1 presents with the highest estimated values among all fibre categories apart from those that are connected to the brainstem. The slopes of FA over time show a different classification of clusters than the intercepts. Clusters 3 and 4 are more similar to each other, while cluster 1 is more similar to cluster 2. Commissural fibres and superficial WM regions show the steepest decline in FA among all the clusters (Fig. 2, fitted FA value maps; Fig. 3, mean FA slopes).

**Table 2:** Demographics of DTI FA clusters

Clustering groups	Cluster 1	Cluster 2	Cluster 3	Cluster 4
Characteristics				
*N*, *N* (%)	186 (45.50%)	63 (15.50%)	74 (18.00%)	87 (21.00%)
Males, *N* (%)	107 (57.50%)	38 (60.30%)	36 (48.60%)	50 (57.50%)
Age, median (mad)	73.0 (10.80)	70.3 (9.60)	75.8 (7.30)	69.4 (8.00)
Education years and median (mad)	16 (3.00)	16 (3.00)	14 (3.00)	14 (3.00)
APOE4 allele carrier, *N* (%)	56 (30.10%)	17 (27.00%)	20 (27.00%)	28 (32.20%)
APOE2 allele carrier, *N* (%)	30 (16.10%)	8 (12.70%)	15 (20.30%)	11 (12.60%)
Health and mean (SD)	2.10 (0.80)	2.40 (0.80)	2.20 (0.90)	2.40 (1.00)
Baseline_CMC, mean (SD)	2.00 (1.40)	2.20 (1.40)	2.10 (1.50)	2.20 (1.50)
Gait speed, median (mad)	113.7 (17.00)	116.30 (17.00)	111.45 (22.00)	113.30 (17.50)
WMH/TIV × 100	0.55* (0.04**)	0.78 (0.07**)	0.83* (0.10**)	1.15 (0.10**)
Amyloid load (PiB SUVR)	1.42** (0.02**)	1.41 (0.02)	1.42 (0.03)	1.40 (0.03)

Diagnosis and cognition																
CU, *N* (%)	168 (90.30%)				53 (84.10%)				66 (89.20%)				71 (81.60%)			
	CU		MCI		CU		MCI		CU		MCI		CU		MCI	
	Male	Female	Male	Female	Male	Female	Male	Female	Male	Female	Male	Female	Male	Female	Male	Female
Global	0.75 (−0.04)	0.54* (−0.01)	0.31 (−0.04)	0.08 (−0.05)	0.25 (−0.04)	1.01 (−0.03)	−0.07 (−0.06)	2.18 (−0.09)	0.58 (−0.03)	1.1 (−0.06)	3.96** (−0.28**)	0.34 (−0.06)	0.29 (−0.04)	0.18 (−0.03)	−0.26 (−0.04)	0.89 (−0.1)
Memory	0.67 (−0.03)	0.49* (0)	−0.53 (−0.02)	0.41 (−0.09)	−0.07** (−0.03)	1.17 (−0.03)	−0.52 (−0.04)	2.62 (−0.15)	0.43 (−0.02)	1.4 (−0.05)	2.42** (−0.21**)	−0.57 (0.01**)	0.09 (−0.01)	0.23 (−0.01)	−0.65 (0)	0.77 (−0.06)
Executive	0.62 (−0.05)	0.82* (−0.03*)	0.51 (−0.07)	−0.48 (0.01)	0.37 (−0.05)	0.93 (−0.05)	0.29 (−0.07)	0.57 (0)	0.42 (−0.03)	1.22 (−0.08)	3.66 (−0.26)	−0.14 (−0.07)	0.5 (−0.07)	0.64 (−0.07)	0.05 (−0.09)	1.19** (−0.15)
Language	0.65 (−0.04**)	0.3 (0)	0.33 (−0.04**)	0.79 (−0.12**)	−0.08** (−0.02)	0.84 (−0.04)	−0.33** (−0.03)	3.91 (−0.17)	0.14 (−0.01)	0.7 (−0.03)	2.13 (−0.16**)	0.05 (−0.04)	0.19 (−0.04)	−0.08 (−0.02)	−0.84 (−0.01)	0.44 (−0.09)
Visuospatial	0.62 (−0.02)	0.34 (−0.01)	0.3 (−0.02)	−0.73 (0.02)	0.6 (−0.03)	0.49 (−0.01)	0.38 (−0.04)	0.11 (0)	0.72 (−0.02)	0.41 (−0.02)	3.56 (−0.21)	0.36 (−0.05)	0.29 (−0.01)	−0.1 (−0.01)	−0.19 (0.01)	0.27 (−0.04)

Main demographic, clinical and biomarker characteristics of the data set (Table 1). The percentages of the clusters sum to 74% of the sample, since a 26% (143 participants) have uncertain classification to any of the clusters (Spplementary Table 1). Health, how healthy the participant feels from 0 to 4 with 4 being worst; CMC, cardiovascular and metabolic conditions; CU, cognitively unimpaired; WMH/TIV × 100 (WMH in FLAIR MRI as a fraction of total intracranial volume; amyloid load, PiB SUVR-Pittsburgh compound B (PiB) PET SUVR] and mad, median absolute deviation. The four domains and their average refer to cognitive functionality, they are *Z*-values and higher is better cognitive score. For WMH/TIV × 100, amyloid load and the cognitive variables (Global, Memory, Executive, Language and Visuospatial), the data is presented as: estimated fitted value at the 60 years of age (estimated annual change after the age of 60 years). The values are estimated with mixed-effect models: Cluster 1 is the reference group for the models of WMH and PIB (reference group for the cognitive models are cluster 1, CU and female), single asterisk (*) signifies significant difference from 0 in intercept or slope of the reference group, whereas double asterisks (**) signify that a group is significantly different from the reference group in intercept or slope (all tests have a 0.05 confidence level and are corrected with the Satterthwaite’s method and corrected for multiple comparisons with the Holm–Šidák method).

The four clusters do not differ in terms of amyloid beta load. All clusters have intercepts around 1.42 PiB standardized uptake value ratio and 0.02 units increase per year (Table 2). WMH as percentage of intracranial volume at baseline were different between the four clusters. However, the mixed-effect model that was used to assess differences between WMH of the four groups revealed that clusters 1–3 have similar intercepts at the age of 60 years and cluster 4 has the highest WMH load, but this estimation was not significant. Regarding the annual increase in WMH, cluster 1 has the lowest rate of WMH accumulation (0.04%), whereas clusters 3 and 4 have significantly higher rate of WMH accumulation (0.1%).

The differences in cognitive profiles between the clusters, expressed in *Z*-values showed that the group of CU subjects, all clusters have similar global cognition intercepts (estimated at the age of 60 years). Males had slightly higher values (Table 2 and Supplementary Fig. 2). Larger differences in global cognition were observed between MCI participants of the four clusters. In cluster 3, males had higher global cognitive intercepts but much steeper decline (0.28 per year) than the other clusters. The steepest decline in memory performance was observed in cluster 3 for MCI (0.21 for males and 0.01 for females per year) compared to the other clusters, whereas sex differences were significant. Executive performance intercepts were similar between clusters for the CU individuals. MCI females of cluster 4 had a higher intercept in executive function. CU males of cluster 2 had lower intercept in language performance. CU males of all clusters had worse decline in language performance than CU females. MCI individuals declined in language performance more than CU individuals in all clusters and MCI males also declined more than females. More specifically, males of cluster 2 started out with worse language performance and males of cluster 3 had very steep estimated decline rates (−0.16 per year). Finally, visuospatial performance did not differ between clusters in estimated intercepts or slopes.

### Relationship between WM tracts and cluster characteristics

**Figure 4:**
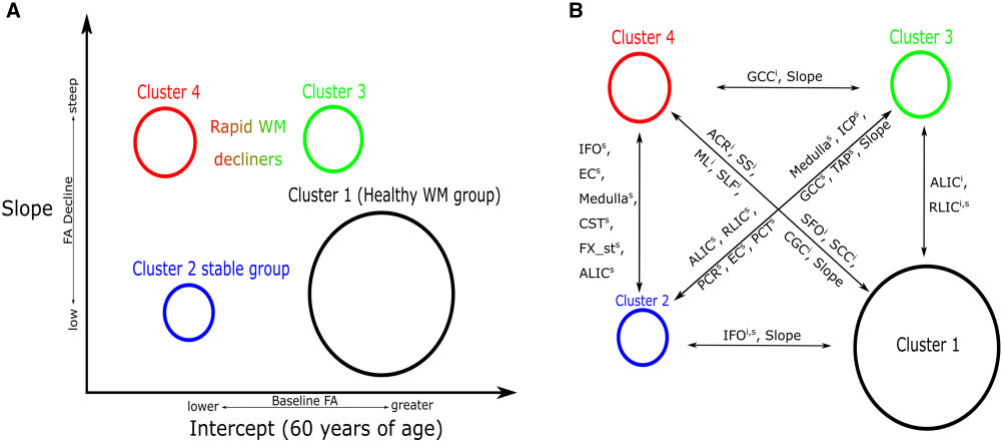
**Cluster WM integrity characteristics.** Cluster WM-specific characteristics are described. (**A**) Cluster intercepts and slopes that were computed by the clustering model. The vertical axis of this figure describes the differences in FA progress over time for the clusters, whereas the horizontal axis describes the intercept differences between them. (**B**) Regions that differ in WM integrity between pairs of clusters. These are the important regions of WM integrity calculated by a post-clustering comparison between clusters of WM integrity. The superscripts *i* and *s* correspond to specific WM tract intercepts and slopes; Slope, a pair of clusters have different slopes. The circle that describes the clusters is proportional to its prevalence in the samples.

The different variables that separated the four clusters can also be visualized with the help of heat-maps (Fig. 3). In terms of intercepts of FA values, cluster 1 was more similar to cluster 3, whereas cluster 2 was more similar to cluster 4. Regarding the slopes of FA over time, the longitudinal information showed that clusters 1 and 2 were more similar to each other and cluster 3 was more similar to cluster 4. There is a distinct difference in the behaviour of WM integrity intercepts in comparison with the grouping of the slopes. The regions that were mostly informative in the discrimination between the clusters included the corpus callosum, the external and internal capsule, uncinate, posterior corona radiata, cingulum (cingulate gyrus), inferior fronto-occipital fasciculus, superior longitudinal fasciculus, medulla and mid-brain WM tracts. For ease of interpretation, we show these findings using Fig. 4B.

**Figure 5:**
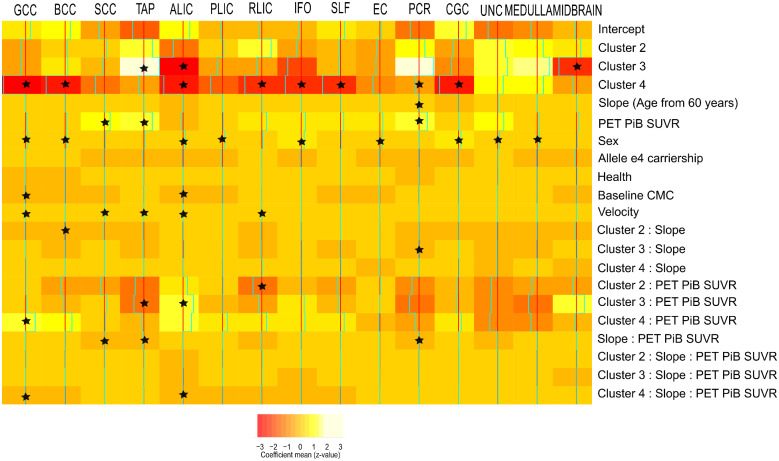
**Relationship between the most discriminant WM integrity fibre tracts and the cluster characteristics.** The colours here show the estimated coefficient of a multivariate multi-output mixed-effect linear regression where WM bundles are output and the different characteristics of the model are used as input. A random intercept was used to account for repeated measures. The asterisk (*) in the cells shows which effects had 95% posterior density intervals that did not include 0, which makes them significant. Reference category is cluster 1, male, non-e4 carrier at baseline. The colours in the colour bar go from red (s.d., −3) to white (s.d., +3).

Finally, Fig. 5 shows the results of the statistical analysis to identify the most discriminative features of the clustering which was used as an output. We used the cluster number as well as some cluster additional characteristics as the input with cluster 1 as the reference group here. We observed similar differences in FA between clusters 1 and 2 as shown in Figs 2 and 3. Sex was associated with FA deterioration in some regions as well as amyloid accumulation. Gait speed showed strong associations with many regional FA values at baseline. Though we did not observe differences in amyloid at the group level, some differences between clusters and amyloid accumulation were observed for a small set of WM tracts FA at baseline (Fig. 5). Longitudinal data showed subtle associations between cluster 4 and amyloid accumulation for two WM tracts (genu of the corpus callosum and anterior limb of internal capsule) in comparison to cluster 1.

## Discussion

The interplay between WM microstructural integrity changes and ageing has been shown to impact cognitive and motor functions. However, most of the conclusions about these relationships are drawn from population level inferences. Investigating whether groups of individuals have different relationships between cognition and WM integrity is of great importance. To enable early prevention of declines in cognition and motor function, it is important to identify individuals who may be vulnerable to WM damage early by studying the deterioration of WM across the population. Capitalizing on (i) unsupervised statistics, (ii) longitudinal WM integrity and (iii) wide range of clinical characteristics, we found groups of individuals who have distinct clinical and WM characteristics and estimated the evolution of the WM integrity trajectories during ageing.

Our main findings were (i) there was differential ageing between fibre classes. The association/brainstem fibres in contrast to commissural fibres were less likely to decline as a function of ageing across all clusters, whereas projection fibres declined more heterogeneously. (ii) We found that the integrity of specific WM fibres (genu of the corpus callosum, anterior limb of internal capsule and posterior corona radiata) can be useful in identifying individuals at risk of future cognitive decline. (iii) We discovered a group (cluster 1) of healthy WM agers who had higher commissural, association and projection fibre FA (at intercept and over time) accompanied by only subtle changes in cognition and WMH with age. (iv) We also found two groups of individuals with initially intermediate WM integrity: a slow decliners group with low initial global FA values but stable over time both in FA and in cognition (cluster 2), and a rapid decliners group that started with spared FA but declined steeply in many fibres as well as in cognition (cluster 3). (v) Finally, we discovered a rapid WM decliners group (cluster 4) with individuals who presented with low overall WM FA and had substantially greater decline in WM integrity, cognition and WMH with age than the other groups.

Our observations about the differential ageing of WM tracts as well as clusters of individuals have provided us with several important insights. Furthermore, the estimation of intercept and slope of WM integrity profiles for the four clusters helped us understand the effect of ageing in WM clusters while considering the initial cluster differences. We have organized the discussion as follows: we begin with the discussion of WM integrity per fibre class to compare our results with existing literature and then discuss cluster-specific WM and cognitive characteristics, which has not been well described to date.

### Projection, commissural, brainstem and association fibres are impacted differentially by ageing

The fibre tracts in the brainstem had low initial FA values for all four clusters in comparison to other fibre types and did not decline significantly with ageing. Our finding is in line with the previous cross-sectional studies that investigated WM degeneration in relation to cognition and found no associations with ageing of brainstem fibres (De Groot *et al.*, 2015; Cremers *et al.*, 2016). The second class of WM fibres that showed marginal decline with ageing were the association fibres. Initial severity differences were observed between clusters, whereas the healthy agers (cluster 1) were relatively unaffected. In comparison to other studies that report FA decreases with ageing in association fibres (Ito *et al.*, 2015; Rieckmann *et al.*, 2016) as well as a correlation between those fibres and cognition (Cremers *et al.*, 2016), we found that these tracts do deteriorate but not as much as other fibre types.

Two association fibre tracts that deteriorate over time for all four clusters are the sagittal stratum and the para-hippocampal part of cingulum which are significantly impacted in ageing. Sagittal stratum is an important WM hub (includes the inferior longitudinal and fronto-occipital fascicles) and associations between decline in ageing and amnestic mild cognitive impairment were reported previously in a cross-sectional study by Liu *et al.* (2013). In another study, the para-hippocampal part of the cingulum showed accelerated longitudinal decline in participants at risk of Alzheimer’s type dementia (Rieckmann *et al.*, 2016) and associations with cognitive memory deficits were also reported (Wang *et al.*, 2012; Ito *et al.*, 2015). We hypothesize that the differences we observed in cognitive decline between clusters may be associated with the decline in these association fibres because WM integrity for these fibres was distinct across clusters.

Projection fibres were one of the two types of fibre tracts that showed strong correlations with ageing independent of cluster allocation. Anterior corona radiata, fornix stria and posterior thalamic radiation declined with ageing for all clusters, even if some of them had initially lower FA values. However, the main exception of no age-related (after 60 years) reduction in WM integrity but low FA values for all clusters was the posterior limb of the internal capsule. These projection fibre-related findings (i.e. lower FA at the age of 60 years and faster declines in specific projection fibres) support the age-related alterations in top-down modulation suggested in the literature (Gazzaley and D’Esposito, 2007; Gazzaley *et al.*, 2008) and may partially explain cognitive decline in memory, language and visuospatial function between all clusters at 60 years and onwards.

Finally, commissural fibres showed systematic declines in FA with ageing for all clusters. This agrees with our literature in WM ageing, where correlations between cognitive decline and frontal/commissural WM integrity deterioration as a function of ageing have been reported (Kennedy and Raz, 2009; Bennett and Madden, 2014; Cremers *et al.*, 2016; Vemuri *et al.*, 2019). Specific focal injury is often seen in the smaller diameter fibres of the corpus callosum. This extent of regional distribution of commissural WM integrity decline was different between the clusters, suggesting that distinct systemic health parameters such as vascular risk factors probably contribute to this heterogeneity.

### Identified clusters have distinct WM spatial trajectories and cognitive decline profiles

Cluster 1 had the highest prevalence in the studied population (45%) and presented with the highest WM FA in most tracts at the baseline observations. Interestingly, not all tracts had high FA at the estimated cluster intercept (60 years old). More specifically, the posterior limb of the internal capsule fibres and the cortico-spinal tract had low FA values (as they were present in other clusters). It is important to note here that reduced intercept WM integrity over all clusters in these fibres may be associated with ageing (reference point, 60 years old), reduced motor and executive abilities as well as worse vascular health in the elderly. Djulejić *et al.* (2016) provide a detailed description of the perforating vasculature to this region and it has been generally understood that the perforating vasculature is more vulnerable to age-related changes (also see Importance of anterior WM structures in cognitive ageing section). We believe that the WM profile of this cluster of individuals (WM per tract mean value) is of great importance. In future applications, we can compare it to the WM integrity profile of any individual at the age span of our data set (60+ years old) and calculate the probability of following the same cognitive trajectory as cluster 1.

The cluster 2 that we discovered differed from cluster 1 in WM intercept. This cluster had much lower prevalence and had a greater number of cognitively impaired participants. Differences between clusters 1 and 2 WM intercepts may be explained by greater initial load of WMH and CMC that can gradually worsen WM integrity over time (Vemuri *et al.*, 2015, 2018; Fuhrmann *et al.*, 2019). Slopes of the clusters 1 and 2 were not very different as shown in Fig. 3. This provides evidence that cluster-2 individuals have worse WM health than cluster 1 and possibly are shifted earlier in brain WM ageing but stable over time. This is in line with the similarities that we found between the two clusters in the cognitive-decline domains assessed (memory, executive, language and visuospatial).

Cluster 4 is a distinct rapid WM decliners group that presented with the highest prevalence of cognitively impaired participants among clusters. CU participants in this group are at risk of declining in cognition. The patterns of WM integrity, WMH and cognitive function of this cluster can be well contrasted with cluster 1 and provide us with the two extremes in terms of WM integrity and its correlate with cognition. Finally, cluster 3 is an intermediate group of individuals with WM profiles between clusters 2 and 4. Interestingly, this group presented with similarities in FA (superficial, commissural, projection and association WM tracts) with cluster 1 at the intercept of the model. However, the rapid decline in WM integrity and cognitive function after the age of 60 years of this cluster makes it comparable to cluster 4. We speculate that this cluster consists of participants who were affected by WM pathology ∼15 years later (Fig. 2) than cluster 4 (i.e. shifted in time) and then presented with more rapid decline in cognition and WMH accumulation. Such differences may be caused by normal vasculature variation (Djulejić *et al.*, 2016) and also selective WM vascular pathology that may not be amyloid specific (Vemuri *et al.*, 2015).

We did not observe amyloid accumulation at the group level, though we did observe subtle correlations between amyloidosis and specific WM tract declines in certain clusters. The lack of differences at the population level is in line with our earlier observation that WM changes may be less influenced in pre-clinical stages of amyloidosis (majority of our population is CU) (Vemuri *et al.*, 2019). We hypothesize that WM health is not affected by amyloidosis in the population but will decline substantially after significant neuro-degeneration is seen in MCI/dementia participants (who were not a large part of this investigation).

### Importance of anterior WM structures in cognitive ageing

Consistent with the literature, we found an anterior–posterior gradient with greater declines in the FA of the anterior WM tracts (anterior corona radiata, genu of the corpus callosum and frontal WM) (Grieve *et al.*, 2007; Gunning-Dixon *et al.*, 2009) across all clusters. This is also supported by the WM retrogenesis hypothesis in cognitive ageing, that late myelinated WM fibres are most vulnerable to age- and disease-related damage (Brickman *et al.*, 2012). This is likely driven by greater sclerotic changes of the frontal lobe medullary arteries seen with ageing and dementia (Furuta *et al.*, 1991). However, there were added variations to the underlying anterior WM deterioration seen across clusters. Specifically by comparing all the cluster differences, cluster 3 was more similar to cluster 1 for commissural and superficial WM FA values (age, 60 years) and also had an anterior WM deterioration trend as other clusters did. However, cluster 3 had significantly greater declines in posterior projection and brainstem fibres, similar to that seen in cluster 4 but not present in clusters 1 and 2. On the other hand, cluster 4 had significant declines both in anterior and in posterior WM. This observation points to a significant feature of ageing—the anterior–posterior WM gradient hypothesis in cognitive ageing, and the possible variation between individuals in the population based on pathological profiles.

### Effectiveness of Bayesian cluster optimization for identifying biologically plausible clusters

The model optimization was achieved quickly due to the large amount of information that balanced well with the number of fixed and random effect parameters in the model. A cohort-based sample with mixed or non-existing pathologies was expected to produce more than one FA components that can be expressed with high probability for the same subject. This is a key strength of the analysis to estimate soft clustering allocations that allow for uncertainty estimation. In this way, we were able to understand the heterogeneity in the data set more accurately, because we do not constrain the algorithm to assign each participant in one cluster even if the individual has a mixture of components’ expression. Although a uni-dimensional result with groups of different stages of FA decline could be expected, the selected model revealed four different patterns of FA values with various slopes over time. All patterns are not completely unrelated with each other since some subjects were classified between two clusters and therefore were excluded from Table 2. Interestingly, these subjects are clustered between clusters 1 and 2 as well as clusters 1 and 3. Almost no subjects fall between very dissimilar clusters (in terms of WH integrity), which means that heterogeneity in the data set was well captured by the model definition. This result points to more sophisticated FA profiles, the study of which can only be revealed with track-specific effects and longitudinal data.

### Limitations, strengths and future work

Though we used a large longitudinal imaging data set for this study, we took a simplistic but robust approach by limiting to linear modelling and excluded individuals who did not tightly belong to a cluster. These excluded individuals from the final clusters described from a central component of defining boundaries and help us delineate the similarities and dissimilarities between clusters. However, the exclusion of a large number of individuals is a limitation for the final interpretation of the clusters since it reduces the statistical power of the between-cluster comparisons. A key strength is the population-based sample of individuals (both cognitively impaired and unimpaired) who allowed the modelling of realistic WM trajectories. However, the longitudinal design of the study allowed for more CU subjects to be included since they had a longer follow-up. Future studies will focus on specific tracts identified in this study and their importance in the development of WM pathologies and cognitive decline.

## Conclusion

Using longitudinal WM microstructural integrity data of 553 participants, we have identified four distinct WM patterns in the population. The mapping of these patterns in our sample provided us with insights about the differential ageing of WM integrity. At the age of 60 years, some individuals may have worse WM health (clusters 2 and 4), whereas other individuals may have greater WM integrity (clusters 1 and 3). Each of these clusters also decline at a different rate than others. This type of classification of individuals has not been reported before in the literature. WM health is informative about future declines in cognition as well as development of WMH burden. Furthermore, we identified key WM tracts that may be important to measure. We believe that current WM health in key WM tracts provides important information that can be utilized to identify older individuals at risk of cognitive decline and prevent WM deterioration in the ageing population.

## Supplementary material

Supplementary material is available at *Brain Communications* online.

## Supplementary Material

fcaa238_Supplementary_Data

## Abbreviations

ACR: anterior corona radiata
ALIC: anterior limb of the internal capsule
AWM: angular white matter
BCC: body of the corpus callosum
CGC: cingulum, cingulate gyrus
CGH: cingulum, hippocampus
CMC: cardiovascular and metabolic conditions
CP: cerebral peduncle
CST: cortico-spinal tract
CU: cognitively unimpaired
DTI: diffusion tensor imaging
EC: external capsule
FA: fractional anisotropy
Fx: fornix (column and body of fornix)
FX_st: fornix (cres)/stria terminalis
GCC: genu of the corpus callosum
ICP: inferior cerebellar peduncle
IFO: inferior fronto-occipital fasciculus
IFWM: inferior frontal white matter
IOWM: inferior occipital white matter
ITWM: inferior temporal white matter
LFOWM: lateral front-orbital white matter
MCI: mild cognitive impairment
MCP: middle cerebellar peduncle
MCSA: Mayo Clinic Study of Aging
MFWM: middle frontal white matter
ML: medial lemniscus
MOWM: middle occipital white matter
MTWM: middle temporal white matter
PCR: posterior corona radiata
PCT: pontine crossing tract
PiB: Pittsburg compound B
PLIC: posterior limb of the internal capsule
PoCWM: post-central white matter
PrCWM: pre-central white matter
PTR: posterior thalamic radiation
RLIC: retrolenticular part of the internal capsule
RWM: rectus white matter
SCC: splenium of the corpus callosum
SCR: superior corona radiata
SFO: superior fronto-occipital fasciculus
SFWM: superior frontal white matter
SLF: superior longitudinal fasciculus
SMWM: supramarginal white matter
SOWM: superior occipital white matter
SPWM: superior parietal white matter
SS: sagittal stratum
STWM: superior temporal white matter
UNC: uncinate fasciculus
WM: white matter
WMH: white-matter hyper-intensities
