# Supplementary material for: Longitudinal deterioration of white-matter integrity: heterogeneity in the ageing population
Source: Brain Commun. 2021 Jan 22;3(1):fcaa238. doi: 10.1093/braincomms/fcaa238 (PMC7884606; doi:10.1093/braincomms/fcaa238)
Supplement: fcaa238_Supplementary_Data [file fcaa238_supplementary_data.docx]

**Supplementary material**

Supplementary table 1. Cluster quality measures for solutions of 2:8 clusters. The data are sorted in terms of the most optimal solution given the three criteria of quality.

| **Number of clusters** | **Percentage of MCMC with low mixing** | **Number of subjects with low classification probability** | **Model deviance** |
| --- | --- | --- | --- |
| 2 | 0,021 | 131 | 44233,56 |
| 4 | 0,023 | 143 | 45997,35 |
| 6 | 0,031 | 125 | 48976,5 |
| 5 | 0,025 | 152 | 47275,68 |
| 3 | 0,024 | 154 | 45116,66 |
| 8 | 0,014 | 139 | 53253,81 |
| 7 | 0,039 | 154 | 50126,21 |

Supplementary table 2. Fractional anisotropy mean intercept and slope for each region and cluster

| Region | Coefficient | Cluster 1 | Cluster 2 | Cluster 3 | Cluster 4 |
| --- | --- | --- | --- | --- | --- |
| Anterior corona radiata | Intercept | 1,453 | 0,606 | 0,550 | -0,134 |
| Anterior corona radiata | Slope | -0,055 | -0,060 | -0,056 | -0,068 |
| Anterior limb of internal capsule | Intercept | 0,790 | 0,382 | -0,360 | -0,296 |
| Anterior limb of internal capsule | Slope | -0,014 | -0,034 | -0,012 | -0,048 |
| Angular white matter | Intercept | 1,155 | 0,473 | 1,258 | 0,189 |
| Angular white matter | Slope | -0,037 | -0,055 | -0,074 | -0,076 |
| Body of corpus callosum | Intercept | 1,260 | 0,556 | 1,356 | 0,083 |
| Body of corpus callosum | Slope | -0,058 | -0,072 | -0,096 | -0,082 |
| Cingulum (cingulate gyrus) | Intercept | 1,091 | 0,241 | -0,034 | -0,822 |
| Cingulum (cingulate gyrus) | Slope | -0,035 | -0,037 | -0,023 | -0,015 |
| Cingulum (hippocampus) | Intercept | 1,176 | 0,530 | 0,422 | -0,332 |
| Cingulum (hippocampus) | Slope | -0,044 | -0,052 | -0,044 | -0,042 |
| Cerebral peduncle | Intercept | 0,342 | 0,217 | -0,200 | -0,575 |
| Cerebral peduncle | Slope | -0,010 | -0,019 | 0,010 | -0,012 |
| Corticospinal tract | Intercept | 0,380 | -0,173 | 0,085 | -0,443 |
| Corticospinal tract | Slope | -0,016 | -0,009 | -0,028 | -0,036 |
| External capsule | Intercept | 0,873 | 0,431 | 0,450 | -0,195 |
| External capsule | Slope | -0,020 | -0,053 | -0,048 | -0,052 |
| Fornix (column and body of fornix) | Intercept | 1,270 | 0,825 | 0,630 | 0,477 |
| Fornix (column and body of fornix) | Slope | -0,062 | -0,053 | -0,054 | -0,059 |
| Fornix (cres)/Stria terminalis | Intercept | 1,417 | 0,851 | 0,741 | 0,228 |
| Fornix (cres)/Stria terminalis | Slope | -0,065 | -0,063 | -0,070 | -0,074 |
| Genu of corpus callosum | Intercept | 1,160 | 0,636 | 0,757 | -0,031 |
| Genu of corpus callosum | Slope | -0,050 | -0,078 | -0,064 | -0,075 |
| Inferior cerebellar peduncle | Intercept | 0,427 | 0,424 | 0,360 | -0,004 |
| Inferior cerebellar peduncle | Slope | -0,022 | -0,031 | -0,043 | -0,042 |
| Inferior fronto-occipital fasciculus | Intercept | 0,685 | 0,124 | -0,032 | -0,729 |
| Inferior fronto-occipital fasciculus | Slope | -0,017 | -0,041 | -0,002 | -0,005 |
| Inferior Frontal white matter | Intercept | 1,382 | 0,643 | 1,377 | 0,427 |
| Inferior Frontal white matter | Slope | -0,060 | -0,068 | -0,093 | -0,122 |
| Inferior Occipital white matter | Intercept | 1,246 | 0,467 | 0,563 | -0,199 |
| Inferior Occipital white matter | Slope | -0,052 | -0,055 | -0,040 | -0,043 |
| Inferior Temporal white matter | Intercept | 1,322 | 0,446 | 0,325 | -0,461 |
| Inferior Temporal white matter | Slope | -0,036 | -0,045 | -0,029 | -0,030 |
| Lateral Fronto- Orbital white matter | Intercept | 0,823 | 0,305 | 1,047 | -0,052 |
| Lateral Fronto- Orbital white matter | Slope | -0,050 | -0,057 | -0,064 | -0,049 |
| Middle cerebellar peduncle | Intercept | 0,540 | -0,161 | -0,202 | -0,457 |
| Middle cerebellar peduncle | Slope | -0,016 | 0,006 | 0,002 | -0,015 |
| Middle Fronto-Orbital white matter | Intercept | 0,924 | 0,202 | 0,807 | -0,053 |
| Middle Fronto-Orbital white matter | Slope | -0,055 | -0,047 | -0,065 | -0,054 |
| Middle Frontal white matter | Intercept | 1,487 | 0,501 | 1,111 | -0,115 |
| Middle Frontal white matter | Slope | -0,052 | -0,040 | -0,071 | -0,076 |
| Medial lemniscus | Intercept | 0,222 | 0,152 | 0,431 | -0,154 |
| Medial lemniscus | Slope | -0,014 | -0,020 | -0,033 | -0,046 |
| Middle Occipital white matter | Intercept | 1,528 | 0,509 | 1,346 | 0,049 |
| Middle Occipital white matter | Slope | -0,056 | -0,053 | -0,056 | -0,060 |
| Middle Temporal white matter | Intercept | 1,536 | 0,478 | 0,724 | -0,249 |
| Middle Temporal white matter | Slope | -0,047 | -0,048 | -0,045 | -0,046 |
| Medulla | Intercept | -0,014 | -0,264 | -0,289 | -0,210 |
| Medulla | Slope | -0,009 | 0,006 | -0,007 | -0,018 |
| Midbrain | Intercept | 0,721 | 0,347 | 0,228 | -0,077 |
| Midbrain | Slope | -0,023 | -0,027 | -0,021 | -0,041 |
| Posterior corona radiata | Intercept | 0,767 | 0,030 | 1,028 | 0,060 |
| Posterior corona radiata | Slope | -0,018 | -0,026 | -0,057 | -0,061 |
| Pontine crossing tract (a part of MCP) | Intercept | 0,506 | 0,139 | 0,697 | 0,258 |
| Pontine crossing tract (a part of MCP) | Slope | -0,022 | -0,019 | -0,039 | -0,040 |
| Posterior limb of internal capsule | Intercept | 0,051 | 0,020 | -0,588 | -0,707 |
| Posterior limb of internal capsule | Slope | 0,019 | -0,007 | 0,013 | 0,005 |
| Posterior thalamic radiation (includes optic radiation) | Intercept | 1,640 | 0,659 | 1,306 | 0,175 |
| Posterior thalamic radiation (includes optic radiation) | Slope | -0,069 | -0,071 | -0,074 | -0,079 |
| Post-Central white matter | Intercept | 1,450 | 0,515 | 1,265 | 0,024 |
| Post-Central white matter | Slope | -0,047 | -0,041 | -0,076 | -0,084 |
| Pons | Intercept | 0,267 | 0,176 | 0,487 | -0,149 |
| Pons | Slope | -0,019 | -0,019 | -0,028 | -0,025 |
| Pre-Central white matter | Intercept | 1,452 | 0,614 | 1,274 | 0,108 |
| Pre-Central white matter | Slope | -0,045 | -0,044 | -0,085 | -0,095 |
| Retrolenticular part of internal capsule | Intercept | 0,937 | 0,386 | -0,050 | -0,455 |
| Retrolenticular part of internal capsule | Slope | -0,013 | -0,025 | -0,026 | -0,032 |
| Rectus white matter | Intercept | 0,929 | 0,439 | 0,253 | -0,106 |
| Rectus white matter | Slope | -0,051 | -0,051 | -0,051 | -0,051 |
| Splenium of corpus callosum | Intercept | 1,212 | 0,588 | 1,509 | 0,392 |
| Splenium of corpus callosum | Slope | -0,059 | -0,076 | -0,092 | -0,104 |
| Superior cerebellar peduncle | Intercept | 0,119 | -0,349 | 0,049 | -0,549 |
| Superior cerebellar peduncle | Slope | 0,000 | 0,003 | -0,005 | -0,008 |
| Superior corona radiata | Intercept | 0,719 | 0,145 | 0,834 | 0,032 |
| Superior corona radiata | Slope | -0,007 | -0,020 | -0,062 | -0,065 |
| Superior fronto-occipital fasciculus (could be a part of ALIC) | Intercept | 0,930 | 0,524 | -0,002 | -0,103 |
| Superior fronto-occipital fasciculus (could be a part of ALIC) | Slope | -0,020 | -0,039 | -0,030 | -0,057 |
| Superior Frontal white matter | Intercept | 1,433 | 0,585 | 0,604 | -0,199 |
| Superior Frontal white matter | Slope | -0,038 | -0,040 | -0,047 | -0,057 |
| Superior longitudinal fasciculus | Intercept | 1,079 | 0,179 | 0,449 | -0,351 |
| Superior longitudinal fasciculus | Slope | -0,017 | -0,022 | -0,044 | -0,054 |
| Supramarginal white matter | Intercept | 1,261 | 0,509 | 1,418 | -0,168 |
| Supramarginal white matter | Slope | -0,042 | -0,063 | -0,094 | -0,072 |
| Superior Occipital white matter | Intercept | 1,487 | 0,545 | 1,794 | 0,151 |
| Superior Occipital white matter | Slope | -0,061 | -0,051 | -0,077 | -0,058 |
| Superior Parietal white matter | Intercept | 1,543 | 0,532 | 1,953 | 0,443 |
| Superior Parietal white matter | Slope | -0,063 | -0,053 | -0,101 | -0,094 |
| Sagittal stratum (includes ILF and IFO) | Intercept | 1,558 | 0,458 | 0,489 | -0,334 |
| Sagittal stratum (includes ILF and IFO) | Slope | -0,052 | -0,049 | -0,046 | -0,044 |
| Superior Temporal white matter | Intercept | 1,565 | 0,502 | 1,068 | -0,281 |
| Superior Temporal white matter | Slope | -0,054 | -0,059 | -0,070 | -0,063 |
| Tapatum | Intercept | 0,807 | 0,096 | 0,942 | 0,028 |
| Tapatum | Slope | -0,040 | -0,045 | -0,059 | -0,058 |
| Uncinate fasciculus | Intercept | 0,427 | 0,199 | 0,133 | -0,286 |
| Uncinate fasciculus | Slope | -0,002 | -0,045 | -0,007 | -0,028 |

Supplementary table 3. Assessment of scanner differences in fractional anisotropy measures

| Region | Scanner 1 vs scanner 2 | Scanner 1 vs scanner 3 |
| --- | --- | --- |
|  | P-value | |
| Anterior corona radiata | 0.9995 | 0.6557 |
| Anterior limb of internal capsule | 1 | 0.2956 |
| Angular white matter | 1 | 0.9993 |
| Body of corpus callosum | 0.9995 | 0.9978 |
| Cingulum (cingulate gyrus) | 1 | 0.7578 |
| Cingulum (hippocampus) | 1 | 0.9996 |
| Cerebral peduncle | 1 | 0.9945 |
| Corticospinal tract | 1 | 0.9967 |
| External capsule | 1 | 0.7915 |
| Fornix (column and body of fornix) | 1 | 1 |
| Fornix (cres)/Stria terminalis | 1 | 1 |
| Genu of corpus callosum | 1 | 0.9995 |
| Inferior cerebellar peduncle | 1 | 1 |
| Inferior fronto-occipital fasciculus | 1 | 0.9975 |
| Inferior Frontal white matter | 1 | 1 |
| Inferior Occipital white matter | 1 | 0.9976 |
| Inferior Temporal white matter | 1 | 0.9283 |
| Lateral Fronto- Orbital white matter | 1 | 0.6508 |
| Middle cerebellar peduncle | 1 | 0.9998 |
| Middle Fronto-Orbital white matter | 1 | 1 |
| Middle Frontal white matter | 1 | 1 |
| Medial lemniscus | 1 | 0.9999 |
| Middle Occipital white matter | 1 | 0.9997 |
| Middle Temporal white matter | 1 | 0.9996 |
| Medulla | 0.9693 | 1 |
| Midbrain | 1 | 0.0569 |
| Posterior corona radiata | 1 | 0.9995 |
| Pontine crossing tract (a part of MCP) | 1 | 0.9784 |
| Posterior limb of internal capsule | 1 | 0.9087 |
| Posterior thalamic radiation (includes optic radiation) | 1 | 1 |
| Post-Central white matter | 1 | 0.9995 |
| Pons | 0.999 | 1 |
| Pre-Central white matter | 1 | 0.9998 |
| Retrolenticular part of internal capsule | 1 | 0.9998 |
| Rectus white matter | 1 | 0.9997 |
| Splenium of corpus callosum | 1 | 1 |
| Superior cerebellar peduncle | 1 | 0.9792 |
| Superior corona radiata | 1 | 0.9996 |
| Superior fronto-occipital fasciculus (could be a part of ALIC) | 1 | 0.9996 |
| Superior Frontal white matter | 1 | 0.7257 |
| Superior longitudinal fasciculus | 1 | 0.9994 |
| Supramarginal white matter | 1 | 0.9999 |
| Superior Occipital white matter | 1 | 0.9672 |
| Superior Parietal white matter | 1 | 0.9999 |
| Sagittal stratum (includes ILF and IFO) | 1 | 0.9983 |
| Superior Temporal white matter | 1 | 0.9999 |
| Tapatum | 1 | 0.9941 |
| Uncinate fasciculus | 1 | 1 |

Linear regressions were used for the comparisons between scanners for the different FA metrics. Sex, APOE and age were added as confounders while the most used scanner was used as reference scanner. P-values corrected for multiple comparisons are presented in this table. The value 1 corresponds to a p-value marginally lower than 1. Results were corrected for multiple comparisons with the Holm–Šidák method.

Supplementary table 4: Time interval between subjects visits

|  | **1st Qu** | **Median** | **Mean** | **3rd Qu.** |
| --- | --- | --- | --- | --- |
| 1st_2nd | 2.07 | 2.46 | 2.35 | 2.69 |
| 2nd_3rd | 1.28 | 1.48 | 1.93 | 2.63 |
| 3rd_4th | 1.22 | 1.23 | 1.39 | 1.38 |
| 4th_5th | 1.35 | 1.35 | 1.35 | 1.35 |

The time interval between visits metrics are reported in years.

Supplementary table 5: Demographics of subjects with equal vs more than 1 visits.

| **Clustering groups** | **Cluster 1** | **Cluster 2** |
| --- | --- | --- |
| Characteristics |  |  |
| N, N(%) | 703 (56%) | 561 (44%) |
| Males, N(%) | 368 (52.3%) | 303 (54%) |
| Age, median(mad) | 73.8 (9) | 72.7 (8.2) |
| Education years, median(mad) | 14.68 (2.6) | 14.72 (2.7) |
| APOE4 allele carrier, N(%) | 192 (27.3%) | 165 (29.4%) |
| Health, mean(sd) | 2.3 (0.9) | 2.2 (0.8) |
| Baseline_CMC, mean(sd) | 2.2 (1.6) | 2 (1.4) |
| Gait speed, median(mad) | 110.02 (25.12) | 112.45 (20.66) |
| WMH/TIV*100, | 1.05 %(1.08) | 1.06 %(1.14) |
| SPM12_PIB_RATIO | 1.57 (0.39) | 1.55 (0.37) |
| Diagnosis and cognition |  |  |
| CU, N(%) | 615 (87.5%) | 492 (87.7%) |
| Global, mean(sd) | -0.05 (1.18) | 0.04 (1.06) |
| Memory, mean(sd) | -0.04 (1.17) | 0.09 (1.14) |
| Executive, mean(sd) | -0.15 (1.20) | -0.09 (1.08) |
| Language, mean(sd) | -0.13 (1.21) | -0.08 (1.08) |
| Visuospatial, mean(sd) | 0.03 (1.02) | 0.13 (1.01) |

Health (how healthy the participant feels from 0 to 4 with 4 being worst), CMC = cardiovascular and metabolic conditions, CU (Cognitively unimpaired), WMH/TIV*100 (White matter hyperintensities in T2 MRI as a fraction of total intracranial volume), SPM12_PIB_RATIO (Pittsburgh compound B [PiB] PET SUVR), mad (median absolute deviation). The last five domains that start with refer to cognitive functionality, they are z values and higher is better cognitive score.

Supplementary table 6: Contingency table between 2-cluster and 4-cluster solutions.

|  | 4-Cluster solution | | | | |  |
| --- | --- | --- | --- | --- | --- | --- |
| 2- Cluster Solution |  | Cluster 1 | Cluster 2 | Cluster 3 | Cluster 4 | Sum |
|  | Cluster 1 | 0 | 11 | 9 | 60 | 80 |
|  | Cluster 2 | 183 | 31 | 48 | 1 | 263 |
|  | Sum | 183 | 42 | 57 | 61 | 343 |

The 2-cluster and 4 cluster solution have the following fractional anisotropy patterns in common: Cluster 1 in 2-cluster solution and cluster 4 in 4-cluster solution, Cluster 2 in 2-cluster solution and cluster 1 in 4-cluster solution.

Supplementary table 7: Demographics of DTI fractional anisotropy 2-cluster solution.

| **Clustering groups** | **Cluster 1** | **Cluster 2** |
| --- | --- | --- |
| Characteristics |  |  |
| N, N(%) | 89 (16%) | 333 (60%) |
| Males, N(%) | 53 (59.6%) | 176 (52.9%) |
| Age, median(mad) | 73.2 (8.6) | 73.3 (8.4) |
| Education years, median(mad) | 14.25 (2.91) | 14.88 (2.74) |
| APOE4 allele carrier, N(%) | 30 (33.7%) | 92 (27.6%) |
| APOE2 allele carrier, N(%) | 8 (9%) | 57 (17.1%) |
| Health, mean(sd) | 2.5 (1) | 2.1 (0.8) |
| Baseline_CMC, mean(sd) | 2.4 (1.5) | 2 (1.4) |
| Gait speed, median(mad) | 107.58 (21.95) | 113.76 (19.68) |
| WMH/TIV*100, | 1,99 (1,72) | 0,83 (0.91) |
| SPM12_PIB_RATIO | 1.6263 (0.444) | 1.5616 (0.375) |
| Diagnosis and cognition |  |  |
| CU, N(%) | 69 (77.5%) | 298 (89.5%) |
| Global | -0.4336 (1.2614) | 0.1272 (1.0257) |
| Memory | -0.2075 (1.2866) | 0.1376 (1.1381) |
| Executive | -0.5041 (1.3909) | -0.0113 (1.0304) |
| Language | -0.4862 (1.1333) | 0.0129 (1.037) |
| Visuospatial | -0.1663 (1.1142) | 0.1819 (0.9828) |

Health (how healthy the participant feels from 0 to 4 with 4 being worst), CMC = cardiovascular and metabolic conditions, CU (Cognitively unimpaired), WMH/TIV*100 (White matter hyperintensities in T2 MRI as a fraction of total intracranial volume), SPM12_PIB_RATIO (Pittsburgh compound B [PiB] PET SUVR), mad (median absolute deviation). The last five domains that start with refer to cognitive functionality, they are z values and higher is better cognitive score.

Supplementary Figure 1: Flowchart of the DTI scans sample selection.


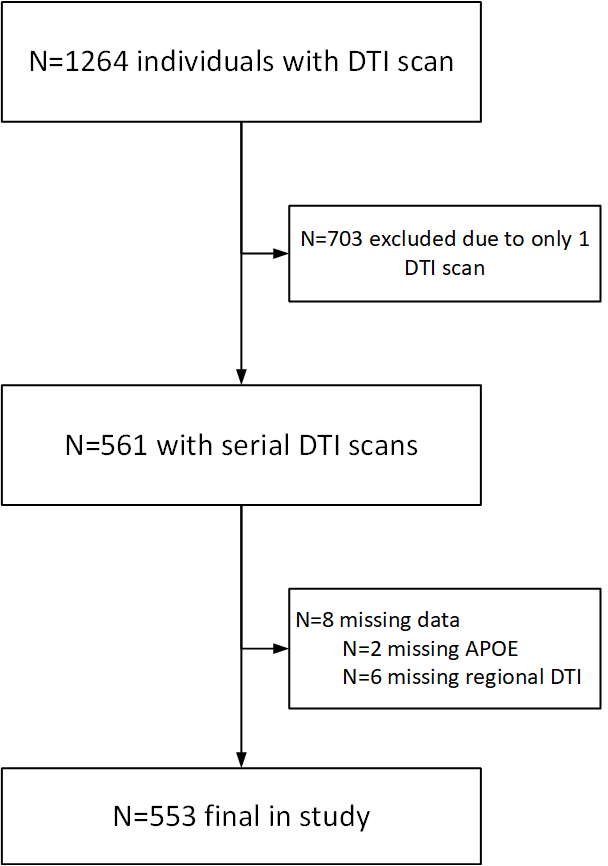


Supplementary Figure 2: Fitted cognitive z-values for WM integrity clusters.

A


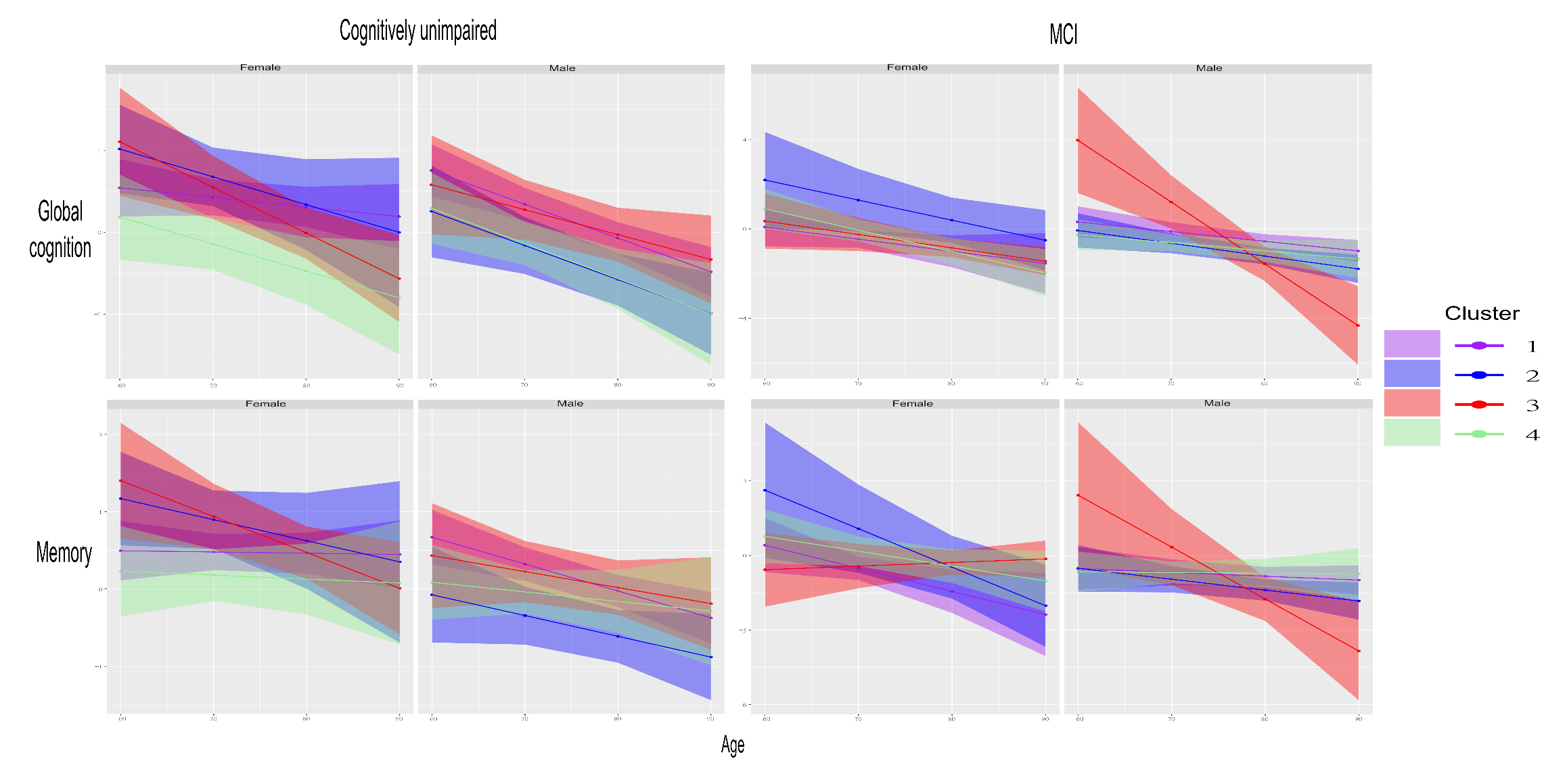


B


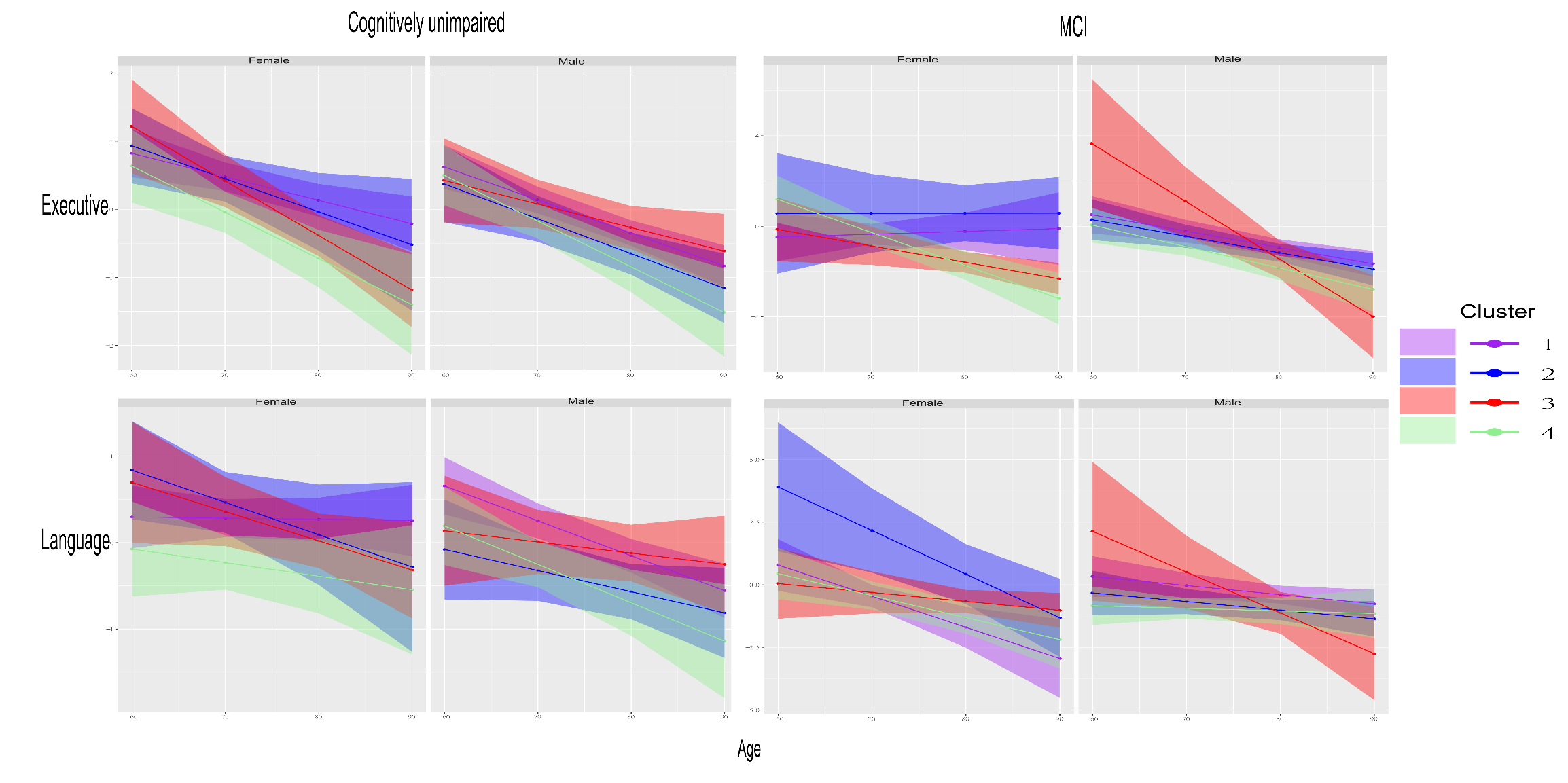


C


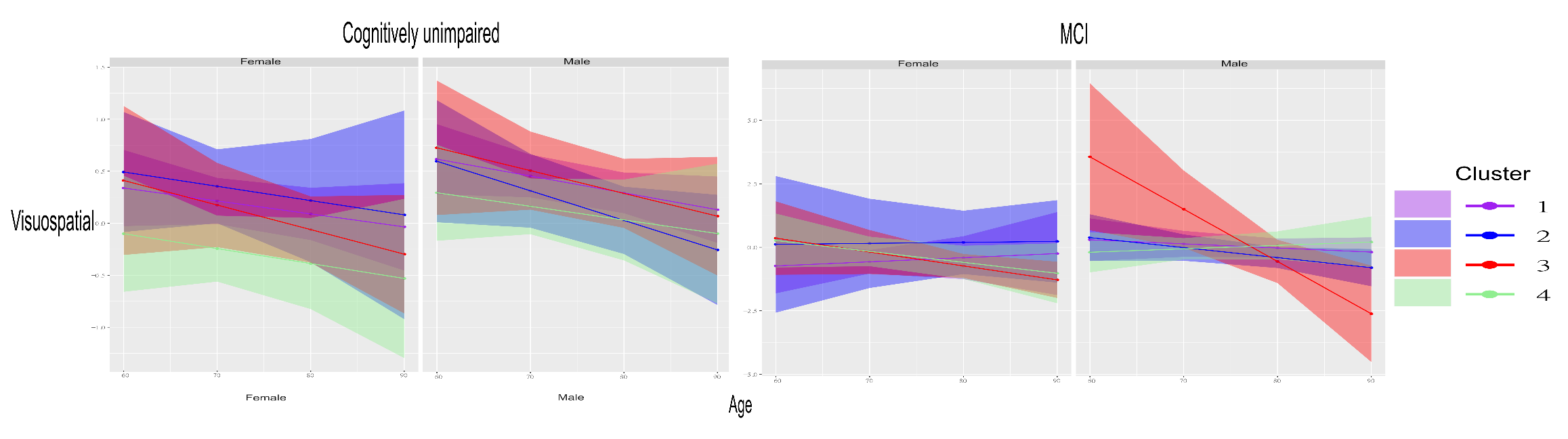


The fitted values were estimated with mixed effect models (one for each cognitive domain). A) Global cognition and memory function, B) Executive and language function, C) Visuospatial function. The model included cognition as response, fixed effects for the interaction between cluster-Age-Sex-diagnosis, subject specific random intercepts.

Supplementary Figure 3: Fitted FA values in different ages for the 2-clusters solution.


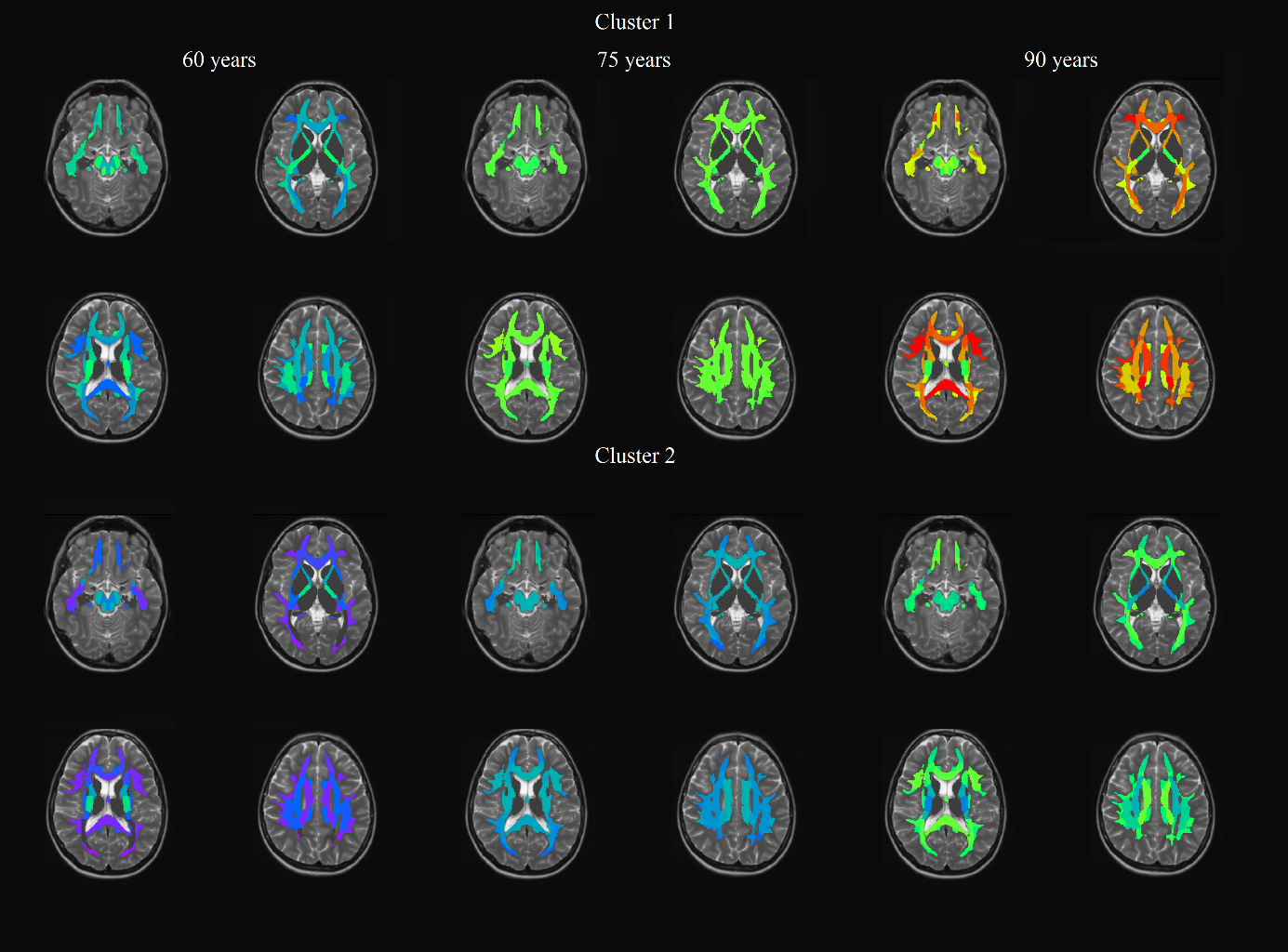


Clustering model fitted values controlled by sex and APOE4 carriership. Fitted values were calculated for the age of 60 years (model intercept), 75 and 90 years. The red colour show lower fractional anisotropy in comparison to yellow colour. The data are z value transformed. The colour scale legend (colour legend in figure 2 of main manuscript) includes 5 colours ordered by increasing fractional anisotropy (red, yellow, green, blue and purple). Clusters are sorted in terms of WM integrity severity trajectories.
